# Pelvic Cellulites

**Published:** 1874-07

**Authors:** D. Colvin

**Affiliations:** Clyde, N. Y.


VOL XIII. JULY, 1874. No. 12.

### Original Communications.

:o:

ART I.—*Pelvic Cellulitis.* By D. COLVIN, M. D., of Clyde, N. Y. Read at the Semi-Annual Meeting of the Central New York Medical Association, December, 1873.

MR. PRESIDENT: I desire to call the attention of this Society to the following cases, not so much perhaps for their rarity,—as I believe they occur far more frequently than they are diagnosed—but to bring to the notice of its members one prominent symptom which I have discovered in each of the cases which I have seen since the literary history of the malady has come to be better understood, as well as to do something to increase their vigilance when such cases come under their supervision and care. I shall, therefore, say but little in relation to the necessary treatment.

I am not reluctant to admit that, in early practice, I met with cases of a similar character, and did not know what I was called upon to treat; but when we understand that usually, by a very little care, there is not much difficulty in ascertaining its true nature, I must confess that, to say the least, I ought to have ascertained the pathological condition of the parts even though the disease had not yet been christened by the name "Pelvic Cellulitis."

The first of the three cases came under my care in the Summer of 1870, and in a family who have but little to do with "Doctors,"

save when the patient is about to die, or until other means (and their name is legion) are exhausted; consequently I did not see her until the case was far advanced.

The patient was a young lady about eighteen years of age, naturally delicate, with a highly sensitive, nervous system.

I ascertained that she was seized with chills, followed by high fever; difficulty in urinating; excruciating pelvic pains, at times extending down the thigh, etc., two weeks previous to my visit. I was informed that the severity of the symptoms was somewhat less, and that I was sent for "to give her something for her ague," also "to strengthen her up." She was much emaciated, and her general aspect indicated one well advanced with hectic symptoms, such as a chill and fever, daily followed by profuse perspiration.

Probably had I been as skilled as Sir Astley Cooper was said to have been on one occasion when in consultation in a case presenting these external symptoms, and when he remarked, the moment he entered the room: "Doctor, where is the matter?" I might at once have instituted a search and found it, as he did, but I did not. I prescribed what seemed to me best calculated to answer the indications, and left, promising to repeat my visit.

At my next visit I found that my patient had not progressed as satisfactorily as I thought she ought under the treatment. In more closely investigating the case, I ascertained that soon after she was taken ill she observed that she could not extend the right leg without increasing the pain, and found it impossible to keep it in an extended position.

That symptom at my first visit was less prominent than it had been. She has also informed me that she fancied as a cause of the illness that she "had taken a little cold during the menstrual flow." I found in the right inguinal region a small tumor, as yet with no distinct fluctuation. For the first time the idea occurred to me that here was a case of Pelvic Cellulitis. I suggested a vaginal examination that my views might be the more fully corroborated.

With much reluctance a consent was obtained. I found a large, slightly fluctuating tumor corresponding exactly with that which presented itself externally. I suggested the propriety of an opening to be made through the vaginal walls, although *then* we had the evidence that it was pointing externally; and, after meeting with opposition to the vaginal opening, was forced to admit that no harm, save delay, would probably arise from waiting a little longer, and promote the external pointing by poultices, etc., when an opening externally could be made.

After much poulticing, I made a free incision into the external tumor, which was followed by an enormous quantity of highly offensive pus. The discharge continued for many days. An active sustaining course soon completed the cure.

My next case was Mrs. N., who had been confined, after her first labor, about ten days previous.

There was nothing unusual connected with the labor, nor during the interval up to the seizure of the symptoms of which I am to speak.

I found, notwithstanding strict injunctions to the contrary, that she had been sitting up too long, and in too low a temperature, the day before. During the night she had a chill lasting so long that the nurse became alarmed, and sent a messenger for me about three A. M.

I saw her immediately, and found that reaction was just setting in. Nothing otherwise, so soon after the chill, presented itself. I prescribed a solution of aconite, promising to see her again in six hours. Being a patient about whom I was unusually anxious, I saw her early in the morning. I found the vascular excitement very high; pulse rapid, although *not hard;* severe pain in the anterior and upper portion of the thigh, lancinating in character, occasionally involving the hypogastric region; slight difficulty in urinating, accompanied with moderate nausea and vomiting.

The following evening there was no great abatement of symptoms, and the vesical difficulty had so far increased as to closely approach complete retention. I hoped, by fomentations, cathartic and anodyne to avoid the necessity of catheterization. Fearing some abdominal inflammation was about asserting itself, I was exceedingly vigilant. The following morning, upon entering the room, I discovered *one* lower extremity, the right, was drawn up. My attention was at once directed to that, not so much from suspecting the true nature of her disease, as that I was looking for peritonitis, metritis, or something of a kindred nature.

I learned that she had passed the night more comfortably than the one previous, but towards morning there was an entire inability to void urine. I also found that she could extend and keep in that position the left leg, but the right was kept flexed from necessity: in other words, the extension of it increased the pain materially. I especially ascertained that it did not produce any pain nor tenderness in the abdominal region generally, still she thought she was "a little more comfortable if the left was flexed *oftener* than was natural." It is unnecessary to say that I catheterized her, but I shall not speak particularly of the rest of the treatment, as that is not the object of this paper.

Suspecting an inflammation of the cellular tissue of the pelvis, I desired a vaginal examination, which was at once had.

I found the roof of the vagina filled with a large, hard, and exquisitely sensitive tumor, pressing firmly upon the bladder, extending, as in my first case, farther to the right side of the cavity.

That this paper may not be too long, consequently taking up too much time of the Society, I will leave the report of this case by saying that so obstinate was this tumor in its progress that I was obliged to catheterize this patient for *thirteen* days—an unusually great length of time. Her pulse became exceedingly feeble and frequent, and notwithstanding the most energetic treatment, believed to be judicious, I feared the case would go on to suppuration, and death be the result.

At the time the bladder began to perform its function, I could discover the beginning of less sensibility in the tumor and a slight reduction in size. I cannot imagine a case where there would be more danger of suppuration than in this, she being naturally delicate from frequent derangements of digestion and leuco-phlegmatic temperament.

Notwithstanding the effusion was completely absorbed, the limb remained rigidly drawn up for a long time, compelling her to use a crutch, followed by a cane for some weeks.

I am convinced that had suppuration taken place in this case, even though the pointing had been in the most favorable direction. I would have been powerless in my efforts to keep her up.

The third case coming under my observation occurred in an adjoining county, the present year, under the care of an intelligent practitioner.

The lady had been confined twenty days previous to my visit, and was seized with a severe and protracted chill, followed by high fever, difficulty in urinating, sharp pains, neuralgic in character, occupying the right iliac region, and extending down the anterior portion of the thigh, twelve days after the labor. I found the symptoms very much like those in my second case, but far less severe. The right leg was kept flexed all the time, although at no time had it been so bad as in the second case, and the fact had escaped the notice of the patient and attendant until their attention had been drawn to it at my visit, when she distinctly remembered suffering more pain upon extending the right leg than when subjecting the left to the same position. Believing there to be a necessity for a vaginal examination that the diagnosis might clearly be made out, I expressed such a desire, which was granted.

I found a tumor occupying the right iliac region principally, extending nearly to the mesial line—not so firm nor sensitive, nor so large as in the first two cases—filled with serum. It had not encroached so far upon the bladder as in my second case, and had not, therefore, caused such extensive functional derangement of that organ.

I saw the case occasionally, and in a few weeks the contents of the tumor were absorbed, and convalescence was fully established.

Some months after the imperfect report of the first two of these cases had been drawn up, I received the work of Prof. Simpson, as published by Appleton & Co. I find a similar delineation of symptoms, much more elaborated, which occurred in my three cases. I will not, however, weary you by referring to what he says on the subject in this invaluable work, except in relation to one or two points. He says: "It occurs at all ages, in both sexes, in puerperal and non-puerperal females. The contents and terminations of the tumor are, first, serum; second, pus; third, coagulable lymph; and fourth, sloughing of the cellular tissue." After the first, should we meet with the other contents, will depend, perhaps, upon what stage of the disease we saw the case, or upon the success with which our treatment meets." Again. "If called to a case where we find a high grade of fever, severe pains in and about the pelvis, more or less dysuria, with or without the drawing up of *one* lower extremity, (the latter symptom depending upon how early you saw the patient,) more or less nausea and vomiting, and especially if these symptoms followed soon after confinement, I should *now* suspect Puerperal Pelvic Cellulitis, and would not be content short of an internal examination, and would surely expect to find a tumor pushing inward the vaginal walls."

He also thinks, as reason would teach, that "whichever limb is more especially drawn up, is an indication that the tumor is pressing more particularly upon the nerves and muscles of that side." He also thinks, for what reason I cannot imagine, that "the latter symptom is more apt to be observed in puerperal cases." It was present in my first case.

He says in reference to the treatment: "Our efforts should be directed with the greatest energy to the control of the inflammatory action, thereby preventing suppuration. If we fail in our effort, soon we shall have a modification of symptoms; the fever, which before had been of a continuous character, now puts on well marked remissions and exacerbations; debility increases, attended with profuse perspirations, and the patient is gradually, and occasionally rapidly, reduced in strength, and comes to present an appearance which it is not necessary to describe, but which is almost pathognomonic of suppuration."

After becoming farther assured, by an examination, that suppuration has taken place, of course an *early* outlet is imperative, that you may divert the direction of the pus rather than allow it to point in a dangerous direction. Although this disease is not usually fatal, there are occasional instances where the cavity does not close, and a continual drain is kept up. At other times, after the discharge has ceased, and the case is seeming to go on to a permanent cure, the discharge returns and soon runs into a chronic form, the patient being quite likely to die of a tubercular disease of the peritoneum.

